# The Poor Man's Lawyer

**Published:** 1897-05-08

**Authors:** 


### Modern Sociology.

### THE POOR MAN'S LAWYER.

Readers of "Redgauntlet"—if in this generation such there be—may remember Peter Peebles, the penniless litigant, who pursued Alan Fairford, the counsel assigned to him to conduct his interminable lawsuit, as if the young advocate had been a runaway slave. To many the incident must have seemed farcical and meaningless, for comparatively few know of the provision which the law of Scotland makes for poor litigants.

Every year a number of lawyers, both writers and advocates (which means, in English parlance, solicitors and barristers), are appointed to act as lawyers for any of the poor who may require their services. Each city or county town appoints from among its own lawyers as many as its size may render necessary. In Glasgow, for example, there are eighteen. The gentlemen appointed to the post are generally young lawyers, anxious more for experience than for reward, of which indeed their chances are few. Of the eighteen, six are appointed to act together for a month at a time, three being assigned to civil and three to criminal work. Thus each has four "turns" in the year, two of each kind of practice.

The civil work is the more interesting. Any would-be litigant has, in the first place, to bring a certificate from some respectable person declaring that he is really unable to pay for the advice he needs. That being accepted, he has assigned to him one of the three young men who are then acting as poor's agents, as they are called. This lawyer acts as his counsel, and, having studied the case, brings it before a "probabilis court." This court consists of the two other young men, who listen to their colleague's statement of the case, and judge from it if his client has a case worth carrying before a sheriff—the judge in minor trials. The judgment of the two who form the probabilis court, so called because it inquires merely whether the litigant has a "probable" case to go on with, is of no legal value. They cannot pronounce a decree; they can only give an opinion. They are, however, allowed to summon witnesses, both for the prosecution and for the defence, and take every means to form an opinion which is reliable and just. If they decide in favour of the complainant, the counsel carries the case into court, and can, if need be, take it from one tribunal to another till it is decided in the supreme court. In turn the counsel in this case is judge in another, and so the youthful lawyer gains experience by investigating these cases, as the youthful doctor gains experience in the hospital.

With the criminal cases things are much simpler. It is the accused only who needs the services of the poor's agent, and, speaking generally, there is a sufficiently probable case against him. But from the accused in these cases no special plea of poverty is demanded, and it sometimes happens that the poor man's counsel is working for someone who is able to pay for his services, but who from neglect, ignorance, or obstinacy has omitted to engage a lawyer to defend him. In the civil court the unpaid advocate usually appears for the prosecution; in the criminal court invariably for the defence; thus he gains experience in all kinds of work, and practises his powers in both accusation and defence. It might be thought that perhaps such untrained services are of little use to the clients; but this is not inevitably so, and, indeed, for the poor and ignorant, who have often difficulty in stating their case, whose vocabulary is limited, and who yet lack brevity as much as precision in their statements, almost any advocate is better than none. And if he be weak in either law or eloquence, the judge before whom he appears—who, perhaps, was himself a poor man's lawyer in his own early days—will often help him out in the statement of a case.

It remains to be said that, though the poor's agent undertakes to work for nothing, he is occasionally successful in getting a fee. Thus the accused in the criminal court, whom his own will, and not his poverty, has induced to take this gratuitous help, may, if he be acquitted and of a grateful sort, remunerate his counsel. In the civil court the client very rarely can pay anything; but if the lawyer gains his case and obtains a judgment against the other party with costs, he may put in his claim for expenses, and be paid for his work. Still, the chief gain of the year's work is the experience it gives, and the varied wrongs and grievances with which he deals as a poor man's lawyer are often valuable training for more remunerative work.

